# The Importance of Lipid Conjugation on Anti-Fusion Peptides against Nipah Virus

**DOI:** 10.3390/biomedicines10030703

**Published:** 2022-03-18

**Authors:** Marta C. Marques, Diana Lousa, Patrícia M. Silva, André F. Faustino, Cláudio M. Soares, Nuno C. Santos

**Affiliations:** 1 Instituto de Medicina Molecular, Faculdade de Medicina, Universidade de Lisboa, 1649-028 Lisbon, Portugal; martamarques@medicina.ulisboa.pt (M.C.M.); patriciamsilva@medicina.ulisboa.pt (P.M.S.); afaustino@ibet.pt (A.F.F.); 2 Instituto de Tecnologia Química e Biológica António Xavier, Universidade Nova de Lisboa, 2780-157 Oeiras, Portugal; dlousa@itqb.unl.pt (D.L.); claudio@itqb.unl.pt (C.M.S.)

**Keywords:** antivirals, peptides, lipid conjugation, Nipah virus, fusion inhibitors

## Abstract

Nipah virus (NiV) is a recently emerging zoonotic virus that belongs to the *Paramyxoviridae* family and the Henipavirus genus. It causes a range of conditions, from asymptomatic infection to acute respiratory illness and fatal encephalitis. The high mortality rate of 40 to 90% ranks these viruses among the deadliest viruses known to infect humans. Currently, there is no antiviral drug available for Nipah virus disease and treatment is only supportive. Thus, there is an urgent demand for efficient antiviral therapies. NiV F protein, which catalyzes fusion between the viral and host membranes, is a potential target for antiviral drugs, as it is a key protein in the initial stages of infection. Fusion inhibitor peptides derived from the HRC-domain of the F protein are known to bind to their complementary domain in the protein’s transient intermediate state, preventing the formation of a six-helix bundle (6HB) thought to be responsible for driving the fusion of the viral and cell membranes. Here, we evaluated the biophysical and structural properties of four different C-terminal lipid-tagged peptides. Different compositions of the lipid tags were tested to search for properties that might promote efficacy and broad-spectrum activity. Fluorescence spectroscopy was used to study the interaction of the peptides with biomembrane model systems and human blood cells. In order to understand the structural properties of the peptides, circular dichroism measurements and molecular dynamics simulations were performed. Our results indicate a peptide preference for cholesterol-enriched membranes and a lipid conjugation-driven stabilization of the peptide α-helical secondary structure. This work may contribute for the development of highly effective viral fusion against NiV inhibitors.

## 1. Introduction

Nipah Virus (NiV) is a recently discovered zoonotic virus that belongs to the genus Henipavirus of the *Paramyxoviridae* family [1]. NiV infection can be asymptomatic, but often leads to acute encephalitis, among other clinical manifestations, including pulmonary disease [2,3]. The high mortality rates of 40 to 75% ranks this virus among the most deadly of those known to infect humans [4,5].

The natural reservoir of Henipavirus genus, which includes NiV and Hendra virus (HeV), are fruit bats of the genus *Pteropus*, commonly known as flying foxes. These pathogens can be transmitted by contact with infected hosts (pigs and horses), consumption of contaminated date palm sap and human-to-human transmission [6,7]. Human-to-human transmission is highly associated with sporadic outbreaks that are responsible for hundreds of human fatalities. One of the most recent outbreaks of Nipah virus infection took place in Kerala, India, in 2018, and was responsible for 19 confirmed cases, including 17 deaths [8,9,10]. Due to its high pathogenicity, NiV infection was designated one of 10 priority diseases posing a public health risk by the World Health Organization Research and Development Blueprint of 2018 [10,11]. NiV disease treatment is mostly limited to supportive care. Monoclonal antibodies for use in human infection, such as the monoclonal antibody m102.4, have been developed for henipaviruses, but this approach is of limited utility in outbreaks [12], due to the expensive nature of antibody-based drugs.

In the last decades, antiviral peptides have attracted attention as a new class of drugs to inhibit viral infections. Antiviral peptides are therapeutic agents that may offer selectivity and specificity, both in vitro and in vivo. Moreover, they display low levels of side effects, making them promising next-generation therapeutic agents [13,14]. This type of drug development approach has been clinically validated for the human immunodeficiency virus type-1 (HIV-1), with the development and approval of the HIV-1 gp41 HRC-derived peptide enfuvirtide (Fuzeon), currently used in the treatment of HIV-1 infected patients [15].

NiV is an enveloped virus and the outer surface of the envelope contains two glycoproteins: the receptor-binding (G) and fusion (F) proteins [16]. NiV infection begins with receptor recognition and attachment by the G protein, leading to the activation of the F protein, which undergoes a conformational rearrangement that drives its hydrophobic fusion peptide into the host cell membrane. The resulting pre-hairpin intermediate structure brings the viral and cell membranes together and, subsequently, the pre-hairpin intermediates of a trimer of F proteins collapse into a fusion-competent structure: the six-helix bundle (6HB), thought to be responsible for driving the fusion of the viral and cell membranes [17,18,19]. During 6HB formation, there is an association between the C-terminal heptad repeat (HRC) and the N-terminal repeat (HRN) of each F protein. Fusion inhibitor peptides may target the transient structure of F either at the HRC or HRN, preventing 6HB formation by interfering with the refolding of the protein [20,21]. Recent studies have shown that peptides derived from the HRC domain of the related human parainfluenza virus type 3 (HPIV3) F protein were able to inhibit membrane fusion by binding to the transiently exposed HRN coiled coil, interfering with the formation of the 6HB [21,22]. It was recently shown by us that the inhibitory potency of HRC-derived fusion inhibitor peptides can be enhanced by the addition of flexible polyethylene glycol (PEG) linkers and through lipophilic moiety conjugation [23].

A set of new lipopeptides with different specific lipid conjugates (namely, cholesterol and tocopherol) was recently reported for its NiV prophylactic efficacy in primates [24]. Using the same set of peptides, we explored the role of the lipid conjugation in the biophysical and structural properties of potential fusion inhibitor peptides, here termed VIKI peptides, and determined how these properties can be correlated with antiviral efficacy. We evaluated the interaction of the VIKI peptides with membranes of different composition and with human blood cells using microscopy and spectroscopy techniques. Additionally, we performed circular dichroism (CD) and molecular dynamics (MD) simulations of the peptide conjugates to characterize their structural properties. Our data indicate that the lipid-conjugated peptides have more affinity with cholesterol-enriched membranes. CD spectroscopy indicated that the lipid conjugation seems to stabilize the peptide α-helical secondary structure, which was further analyzed by MD simulations.

## 2. Materials and Methods

### 2.1. Chemicals and Reagents

NaCl, L-tryptophan (Trp), dimethyl sulfoxide (DMSO), sodium citrate, chloroform and acrylamide were purchased from Merk (Darmstadt, Germany). HEPES (4-(2-hydroxyethyl)-1-piperazine ethanesulfonic acid), cholesterol (Chol), tocopherol (Toco), Pluronic-F127, ANS (8-anilino-1-naphthalenesulfonic acid), 5-NS (5-doxyl-stearic acid) and 16-NS (16-doxyl-stearic acid) were acquired from Sigma-Aldrich (St. Louis, MO, USA). The phospholipid 1-palmitoyl-2-oleoyl-sn-glycero-3-phosphocoline (POPC) was purchased from Avanti Polar Lipids (Alabaster, AL, USA). The fluorescence probe di-8-ANEPPS (4-(-(6-(dioctylamino)-2-naphthalenyl)ethenyl)1-(3-sulfopropyl)-pyridinium) was purchased from Invitrogen–Molecular Probes (Eugene, OR, USA). Lymphoprep was obtained from Stemcell Technologies (Vancouver, BC, Canada). The n-dodecyl β-maltoside (DM) was purchased from GLYCON Biochemicals (Luckenwalde, Germany). Large unilamellar vesicles (LUVs) were prepared by manual extrusion, as described elsewhere [25,26].

### 2.2. Peptides

This work involved five peptide conjugates, derived from the same VIKI peptide sequence: VALDPIDISIVLNKIKSDLEESKEWIRRSNKILDSI, previously designed and shown to inhibit viral entry [22]. The five peptides studied, generally denominated here as VIKI peptides, are defined as: VIKI-dPEG_4_ for VIKI–GSGSG-C-dPEG_4_–Cys–(CH_2_CONH_2_); VIKI-dPEG_4_-Chol for VIKI–GSGSG–C-dPEG_4_–Cys–cholesterol; VIKI-dPEG_4_-Toco for VIKI–GSGSG–C-dPEG_4_–Cys–tocopherol; VIKI-dPEG_4_-bisChol for VIKI–GSGSG–C-dPEG_4_–Cys–(PEG_4_-bis-cholesterol); and VIKI-dPEG_4_-bisToco for VIKI–GSGSG–C-dPEG_4_–Cys-(PEG_4_-bis-tocopherol). Peptides were purchased with >95% purity from American Peptide Company (Sunnyvale, CA, USA) and received under the framework of NIH project R01AI114736, lead by Anne Moscona (Columbia University Medical Center, NY, USA). Purity and molecular masses were evaluated by mass spectrometry, HPLC and amino acid sequencing. Due to the low solubility of the lipid-tagged peptides in aqueous buffer, peptide stock solutions (500 μM) were generally prepared in DMSO and stored at −20 °C, unless otherwise stated. The final DMSO concentration on experiment conditions was always kept under 2% (*v*/*v*), unless otherwise stated. The peptides’ concentrations were calculated according to the absorbance at 280 nm, by using a molar absorption coefficient (ε) of 5690 cm^−1^M^−1^.

### 2.3. Fluorescence Spectroscopy

Steady state fluorescence spectroscopy measurements were conducted at room temperature in a Varian Cary Eclipse fluorescence spectrophotometer (Mulgrave, Australia), except for the ANS measurements, which were performed in an Edinburgh Instruments FLS920 fluorescence spectrophotometer (Livingston, UK). For the time-resolved fluorescence spectroscopy studies, we used a LifeSpec II Fluorescence Lifetime spectrometer (Edinburgh Instruments, Livingston, UK), equipped with a pulsed excitation laser of 280 nm (vertically polarized). Fluorescence emission was acquired at 350 nm, with 20 nm bandwidth (polarized at magic angle, 54.7°), operating with 20 ns time span and 1024 channels in a multichannel analyzer. Fluorescence intensity values were corrected for dilution and background noise.

All experimental conditions were measured independently and in triplicate. For the average values, the propagated standard error (SE) was calculated as the standard deviation (SD) divided by the square root of the number of points used for the calculation. The SE of *I*/*I*_0_ was calculated according to:

(1) $${\mathrm{SE}}_{\mathrm{ratio}}=\left|\frac{I}{I_{0}}\right|\times \sqrt{{\left(\frac{{\mathrm{SE}}_{I}}{I}\right)}^{2}+{\left(\frac{{\mathrm{SE}}_{I_{0}}}{I_{0}}\right)}^{2}}$$

For the slopes, SE was obtained directly from the error in the calculation of slopes through the fitting of Equation (1) to the data, using GraphPad Prism v. 6 Software (San Diego, CA, USA), using the least squares’ method. The significance of the difference between slopes from residues with normed fit index (NFI) below 0.5 and slopes from residues with NFI above 0.5 was also analyzed using GraphPad Prism v. 6, by employing a two-tailed unpaired *t*-test with Welch’s correction (not assuming equal variances).

### 2.4. Characterization of VIKI Peptides in Solution

UV–Vis absorption and fluorescence (both excitation and emission) spectral characterization of VIKI peptides were carried out in 10 mM HEPES with 150 mM NaCl, pH 7.4. The fluorescence emission spectral shift was used to monitor peptide–DM and peptide–LUV binding, as no significant variations of intensity (quantum yield) were observed. The fluorescence spectral shifts were analyzed by monitoring the alterations in the spectral center of mass (<*ν*>), calculated as:

(2) $$\frac{1}{<\upsilon >}=\frac{\Sigma {\frac{1}{\lambda }}_{i}I_{i}}{\Sigma \;I_{i}}\;\overset{}{\Leftrightarrow }\;<\lambda >=\frac{\Sigma I_{i}}{\Sigma \left(\frac{I_{i}}{\lambda_{i}}\right)}$$

where *I_i_* is the fluorescence emission intensity at wavenumber *ν_i_*, and the summation is performed over the range of measured *I* values. The VIKI peptides’ concentration used in these assays was 10 μM. The micelle stock solution’s concentration was 5 mM DM in HEPES buffer to 2 μM as final concentration. All experiments were performed at room temperature.

### 2.5. Partition Studies

Membrane partition studies were performed by successive additions of small amounts of LUV suspensions with two different lipid compositions, pure POPC and POPC: cholesterol (2:1), to solutions containing 5 μM of each VIKI peptide, independently. There was an incubation time of 10 min between addition of LUV and measurements, as previously recommended [27]. Intrinsic fluorescence measurements of VIKI peptides and Trp were performed with an excitation wavelength of 280 nm and emission between 310 and 450 nm, with excitation and emission bandwidths of 5 and 10 nm, respectively, as reported elsewhere [27]. All conditions were tested independently and in triplicate.

### 2.6. Peptide Aggregation Assessed by ANS Fluorescence

The effect of peptide concentration on its aggregation propensity was followed by means of ANS fluorescence, with excitation at 369 nm and emission collected between 400 and 600 nm, using excitation and emission slits of 5 and 10 nm, respectively. A solution containing 12.8 μM ANS was titrated with a stock solution of the selected peptide to yield an experimental peptide concentration in the 0–8 μM range. The final DMSO concentration was kept under 1.6%. After each peptide addition, the sample was incubated for 10 min before measurement. The fluorescence emission intensity values were corrected for dilution and background noise. All conditions were measured independently and in triplicate.

### 2.7. Fluorescence Quenching

For the acrylamide quenching experiments, an excitation wavelength of 290 nm was used, with spectral bandwidths of 5 nm for excitation and 10 nm for emission, as previously described [27]. All the experimental assays were conducted in triplicate. The fluorescence quenching data were analyzed according to the Stern–Volmer equation for dynamic quenching [28]:

(3) $$\frac{I_{0}}{I}=1+K_{SV}\left[Q\right]$$

where *I*_0_ and *I* are the fluorescence intensities in the absence and presence of the quencher, [*Q*] is the quencher concentration and *K_SV_* is the Stern–Volmer constant [28].

Time-resolved intensity decays were measured when using the lipophilic quencher probes 5-NS and 16-NS, as previously described [26,29]. These assays were performed with the same peptide and lipid concentrations as those used for the acrylamide quenching, by successive additions of the quenchers (in ethanol stock solutions) to 0.5 mM POPC LUVs, after a 10 min incubation, ensuring ethanol concentration below 2% (*v*/*v*) to avoid lipid bilayer perturbations. Positive deviations from linearity in the Stern–Volmer plots were analyzed using the quenching sphere-of-action model [29]:

(4) $$\frac{I_{0}}{I}=1+K_{SV}\left[Q\right]e^{V\left[Q\right]}$$

where *V* is the static quenching constant. In the case of dynamic quenching, the relationship *I*_0_/*I* = *τ*_0_/*τ* is valid; therefore, time-resolved quenching data can be analyzed using Equation (4). All conditions were tested independently and in triplicate.

### 2.8. Membrane Dipole Potential Sensing

Human blood samples were obtained from healthy volunteer donors, with their previous written informed consent, at Instituto Português do Sangue e da Transplantação (IPST; Lisbon, Portugal). Samples were collected into K_3_EDTA anticoagulant tubes (Vacuette, Greiner Bio-One, Kremsmünster, Austria). This study was approved by the Joint Ethics Committee of Faculdade de Medicina da Universidade de Lisboa and Centro Hospitalar Lisboa Norte (Lisbon, Portugal). Isolation of erythrocytes and human peripheral blood mononuclear cells (PBMCs), as well as the labelling of these cells with di-8-ANEPPS, were performed as previously described [27]. Excitation spectra were recorded between 400 and 550 nm, with emission set to 670 nm (excitation and emission bandwidths of 5 and 10 nm, respectively), which avoids membrane fluidity-related artefacts [30,31]. Then, the ratio of intensities (R) at excitation wavelengths of 455 and 525 nm was calculated in order to monitor membrane dipole potential changes. The variation of R with increasing peptide concentration was investigated with a single binding site model [32], as described elsewhere [12].The final DMSO concentration was kept at 1.4% and 1.6% for cells and LUV, respectively. All conditions were tested independently and in triplicate.

### 2.9. Circular Dichroism

Far-UV circular dichroism (CD) spectroscopy data were acquired on a Jasco J-815 CD spectropolarimeter (Hachioji, Tokyo, Japan) using quartz cuvettes of 1.0 mm (Hellma, Müllheim, Germany). All spectra were collected at 25 °C, between 195 and 260 nm, with sampling velocity of 200 nm min^−1^, data pitch of 0.5 nm, data integration of 1 s and 1 nm bandwidth. Each spectrum represents an average of three scans. Peptide stock solutions (500 μM of peptide in 10 mM HEPES at pH 7.4 with 150 mM NaCl and 5 mM DM) were diluted to a final peptide concentration of 20 μM in 10 mM sodium phosphate buffer pH 7.4, with 150 mM NaCl, in the presence and absence of 2 mM POPC LUV and, importantly, in the absence of DMSO. The DM concentration (0.2 mM) was always kept under the critical micellar concentration (CMC) to avoid lipid vesicle leakage. Absorbance spectra were also monitored to control light scattering and signal detection saturation. In addition to blank subtraction, experimental instrument-related baseline drift was corrected by subtracting from each spectrum the average of the signal between 250 and 260 nm. The raw ellipticity, θ, was converted into the mean residue molar ellipticity ([θ], in deg cm^2^ dmol^−1^ Res^−1^). All conditions were tested independently and in triplicate.

### 2.10. Dynamic Light Scattering

Dynamic light scattering (DLS) spectroscopy measurements were conducted on a Malvern Zetasizer Nano ZS (Malvern, UK) with backscattering detection at 173°, equipped with a He-Ne laser (λ = 632.8 nm), at 25 °C, using low-volume 0.3 cm quartz cells (Hellma, Müllheim, Germany). Pure POPC LUVs were diluted to a lipid concentration of 0.5 mM in the same buffer as used for the CD experiments. The size of the LUV was first measured and then DM detergent was added to the lipid vesicles to a final concentration of 2 μM. Samples were left equilibrating for 15 min at 25 °C before each measurement set (30 measurements of 10 s each). VIKI peptides were previously diluted from a 100% DMSO stock solution in PBS buffer, with concentrations ranging from 10 to 20 μM (always under 2% DMSO), with and without sonication for 1 min and vortexing at high speed for 1 min, immediately before new measurements. All buffers were filtered through sterile 0.45 μm pore size filters (Whatman, Florham Park, NJ, USA). Normalized intensity autocorrelation functions were analyzed using the CONTIN method [33,34], yielding a distribution of hydrodynamic diameters (D_H_). For each measurement, D_H_ values were obtained from the peak of the scattering particle number distribution.

### 2.11. Molecular Dynamics Simulations

Molecular dynamics’ (MD) simulations were performed to provide molecular insights into the interaction of the lipid-tagged peptides with a membrane bilayer and to analyze the effect of oligomerization on the peptides’ stability. Four different sets of MD simulations were performed: VIKI-dPEG_4_-Chol monomer in water; VIKI-dPEG_4_-Chol dimer in water; VIKI-Chol monomer in the presence of a POPC membrane; and VIKI-dPEG_4_-Chol dimer in the presence of a POPC membrane.

The simulations were performed with the GROMACS package v. 5.0 in the NPT ensemble, at 300 K and 1 atm. A cut-off of 1.0 nm was used for electrostatic interactions and the Particle Mesh Ewald method was used to treat interactions beyond this cut-off. Van der Waals’ interactions were calculated up to 1.0 nm. Peptide and lipid bonds were constrained with the LINCS algorithm and SETTLE was used for water.

GROMOS 54A7 parameters were used for the peptide and POPC molecules and the SPC model was applied to water. Cholesterol parameters were taken from a manually curated topology available in the Automated Topology Builder and Repository, and PEG parameters were based on the GROMOS 53A6_OXY+D_ force field for vicinal diethers. Since the cholesterol group is bonded to the side chain of a cysteine residue, we had to parameterize the region connecting the two moieties. This region extends from the Cβ carbon of the cysteine to the ring carbon of cholesterol that is attached to the oxygen atom. The partial charges of these atoms were calculated by Restrained ElectroStatic Potential (RESP) fitting on electrostatic potentials calculated with GAUSSIAN09 (Gaussian, Inc., Wallingford, CT, USA) (Frisch, M.J. et al., 2009). The parameters for the bond, angle and dihedral interactions were adapted from GROMOS 54A7 force field. In order to increase the sampling efficiency, all the heavy atoms masses were scaled by a factor of 1/10. We note that this procedure does not affect the thermodynamic properties of the systems under study.

The starting structure of the VIKI peptide was obtained by homology modelling, using the crystal structure of the chimeric 6HB formed by the NiV/HeV HRN segment N42 and the HPIV3 HRC segment [22]. This structure contains the wild-type peptide from which the VIKI peptide was derived. The program Modeller v. 9 was used to generate the model. The final model was the one with the lowest value of the objective function out of 20 generated models. In the model obtained, the peptide adopts a continuous α-helix, with the flanking residues in a random coil conformation. The structure of the PEG-Chol tail was built and appended to the VIKI peptide using PyMOL. To build the dimer, two peptide monomers were placed at interaction distance with the hydrophobic residues located at the dimer interface, using PyMOL [35]. All the acidic and basic residues were considered charged and the N- and C-terminals were capped by an acetate and an amine group, respectively.

For the simulations in pure water, the peptide (either in a monomeric or dimeric state) was placed in the center of dodecahedral box, with a minimum distance of 1.0 nm between the peptide and the box walls. This box was then filled with pre-equilibrated water molecules. For the simulations in the presence of a membrane, a system containing the VIKI-dPEG_4_-Chol peptide (either in a monomeric or dimeric state) and a pre-equilibrated bilayer with 512 POPC molecules was built. The cholesterol tail was placed at the entrance of the lipid bilayer, with the tip inserted in the membrane. This system was then solvated with water.

The energy of the systems was minimized in three steps using the steepest descent algorithm. In the first step, the positions of all the heavy atoms were restrained with a force constant of 1 kJ/mol Å^2^. In the second step, only Cα atoms were restrained with the same force constant and, in the last step, no restraints were used. An initialization procedure comprising three steps was carried out to start the MD simulations. In the first 50 ps of simulation, all the heavy atoms were restrained and the simulation was performed in the NVT ensemble with a temperature coupling constant of 0.05 ps^−1^. This was followed by 50 ps of simulation in the NPT ensemble with temperature and pressure coupling constants of 0.05 and 0.5 ps^−1^, respectively, maintaining the restraints on the heavy atoms. In the following 50 ps of simulation, only the Cα were restrained and the temperature coupling was changed to 0.1 ps^−1^. Finally, a simulation comprising 1 ns was performed, in which the residues flanking the VIKI peptide and the PEG-Chol tail were unrestrained, maintaining the same conditions that were used in the previous step. This was carried out to allow the cholesterol tail to find a stable arrangement. After this initialization procedure, an unrestrained simulation was running for 100 ns in the NPT ensemble, at 300 K and 1 atm, using the V-rescale and Parrinello–Rahman coupling schemes for the temperature and pressure, respectively. The temperature coupling constant used was 0.1 ps^−1^ and, for the pressure, a coupling constant of 2 ps^−1^ was assigned.

## 3. Results

### 3.1. Lipid-Conjugation Targets the Peptides to the Membrane

**Figure 1:**
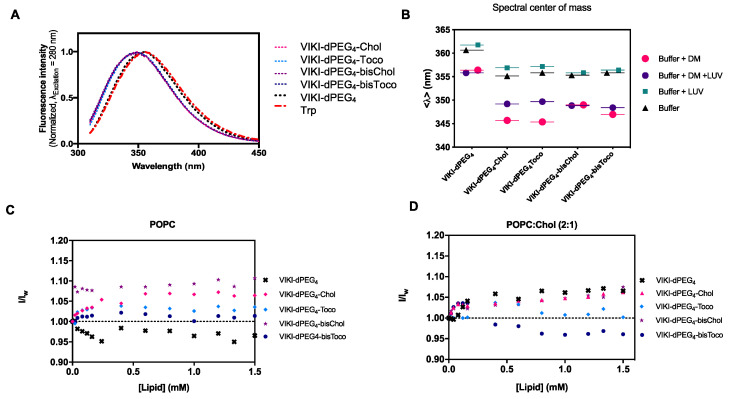
(**A**) Normalized fluorescence excitation spectra of 5 μM VIKI-dPEG_4_, VIKI-dPEG_4_-Chol, VIKI-dPEG_4_-Toco, VIKI-dPEG_4_-bisChol, VIKI-dPEG4-bisToco, and tryptophan in HEPES buffer with 1% DMSO. (**B**) Spectral center of mass (see Equation (2)) of the fluorescence emission variations shown for the VIKI peptides solubilized in DM detergent (circles) in the presence or absence of POPC vesicles (LUV). Peptide-DM monomers and peptide-POPC vesicles interactions can be measured by finding the spectral center of mass of the fluorescence emission. Partition of the fusion inhibitors peptides to POPC (**C**), and (**D**) POPC:Chol (2:1) LUV. All data resulted from mean values from at least three independent experiments.

Peptide–membrane interactions can be assessed by monitoring alterations in the intrinsic fluorescence of the peptide’s aromatic amino acid residues [36]. We first followed the intrinsic Trp fluorescence to assess changes in the solvent exposure of Trp residue present in VIKI peptides, due to interaction with DM monomers and POPC LUV. The VIKI untagged peptide exhibited a maximum emission wavelength at 356 nm when free in solution (Figure 1A). In contrast, the VIKI-derived lipid-tagged peptides presented a shift to the blue (349 nm), indicating that the Trp residue of the lipid-tagged peptides is in a more hydrophobic environment in the same conditions.

In another assay, we used 2 μM of DM detergent to solubilize the peptides in aqueous solution, in the absence and presence of 2 mM of POPC vesicles. We calculated the spectral center of mass and compared the results obtained, which enabled the characterization of the peptide-detergent and peptide-LUV interactions. The lipid-tagged peptides have similar behaviors in buffer and DM monomers, but a fluorescence emission red shift can be observed when POPC LUVs are added (Figure 1B). We also explored the biophysical properties of the lipid-conjugated peptides that affect their membrane insertion. Membrane partition is particularly relevant, especially for the many molecules that have biological membranes as their target [15]. Given that the fusion proteins are bound to the viral membrane, the preferential location of fusion inhibitor peptides at the membrane may facilitate their interaction with the fusion protein during the adoption of its fusogenic conformation. VIKI peptides showed only a small increase in fluorescence intensity in the presence of LUV from both lipid systems, indicating an absence of interaction with the membrane, or an interaction in which the Trp residue is not involved (Figure 1C,D).

**Figure 2:**
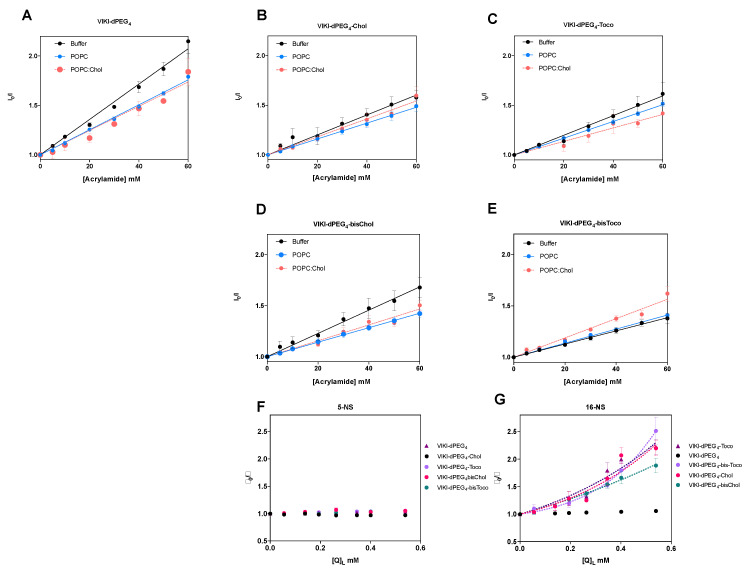
Accessibility of the peptide to the aqueous medium. Fluorescence quenching by acrylamide of VIKI series (**A**–**E**). Lipid compositions tested were pure POPC and POPC:Chol (2:1), using 5 μM peptide and 0.5 mM total lipid. Continuous and dashed lines are fittings of the Stern–Volmer equation. Fluorescence quenching by 5-NS (**F**), and 16-NS (**G**), determined by time-resolved fluorescence spectroscopy in the presence 0.5 mM POPC LUV. Dashed lines are fittings of Equation (3). All values are mean values from at least three independent experiments.

To further evaluate the accessibility of the peptides’ Trp residue to the aqueous environment and complement the membrane partition assays, we used acrylamide as an aqueous quencher, due to its low capacity for penetration into lipid bilayers [29,37]. The LUV tested were composed of pure POPC or a mixture of POPC with cholesterol (33%). In aqueous solution, all the peptides showed linear Stern–Volmer plots (Figure 2). The K_SV_ decreased in the presence of the lipid phase, except for VIKI-bisChol (Table 1), which may indicate a decrease in the fluorescence lifetime of the excited state. Nevertheless, the K_SV_ values in the presence of 0.5 mM LUV are still high, indicating that the Trp residue is sufficiently exposed to the aqueous environment to be quenched by acrylamide. This indicates that the interaction with the membrane occurs with the fluorophores partially exposed to the aqueous environment. In agreement with the results of the partition studies, the peptidic portion, which harbors the Trp residue, does not seem to interact directly with lipids and is probably exposed due to the peptides’ conformation at the membrane surface.

Fluorescence quenching measurements were also performed with lipophilic quenchers in an attempt to assess the depth of insertion of the peptides’ Trp residue inserted into the POPC vesicles. Stearic acid molecules derivatized with a doxyl quencher group either at carbon 5 (5-NS) or 16 (16-NS) were used as lipophilic probes. Figure 2F,G show the Stern–Volmer plots obtained for the different peptides with 0.5 mM POPC LUV, using the effective concentration of 5-NS and 16-NS in the bilayer matrix. In the time-resolved fluorescence experiments using 16-NS, data presented in Figure 2G were analyzed with the sphere-of-action model (Equation (4)), from which a value for the static quenching constant, V, can be recovered (Table 2).

The K_SV_ values for static quenching show that most of the peptides should rest in the vesicles’ surface. As a result, fluorophore quenching by 5-NS was not efficient, when compared with 16-NS. Fluorescence lifetime quenching data obtained does not permit the application of the SIMEXDA method [38] to quantify the depth of insertion of the Trp residues in the membrane, since there is no quenching by 5-NS.

The results indicate that the lipid-conjugation strategy targets the peptides to the membrane and that is the lipid portion (cholesterol or tocopherol), which is responsible for the membrane anchoring. 

### 3.2. Bis-Tagged Peptides Display Membrane-Increased Affinity

Membrane binding emerged as an important factor to take into account concerning the mode of action of these peptides [28]. We made use of an indirect reporter, the fluorescent lipophilic probe di-8-ANEPPS. This probe is incorporated in the outer leaflet of liposome and cell membranes. A fluorescence red shift is expected, which is an indication of a decrease in the membrane dipole potential in a peptide concentration-dependent manner. To be able to quantify this interaction, we measured the ratio of the intensities at the excitation wavelengths 455 and 525 nm, with emission at 670 nm (R) for a range of peptide concentrations. R is a quantitative descriptor of spectral shifts and, hence, of the relative variation of dipole potential.

**Figure 3:**
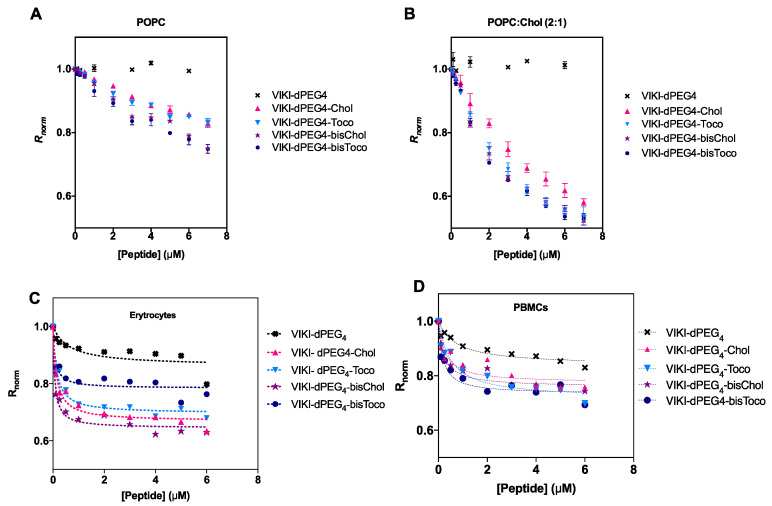
Membrane binding profiles of the VIKI-peptides to (**A**) POPC and (**B**) POPC:Chol (2:1) LUV, and to (**C**) erythrocytes and (**D**) PBMCs, assessed using the fluorescence probe di-8-ANEPPS. Binding profiles were obtained by plotting the di-8-ANEPPS excitation ratio, R (I_455_/I_525_, normalized to the initial value), as a function of peptide concentration. DMSO, cholesterol and tocopherol were also tested, as controls, and no significant changes in dipole potential were observed (data not shown).

The membrane dipole potential decreased in the presence of all lipid-tagged peptides, when compared to the untagged, as shown in Figure 3. These results are consistent with previous studies that show that the lipid-tagging strategy enhances the peptides’ affinity to membranes [27]. As indicated in Figure 3B, there is a preference for cholesterol-enriched vesicles. 

We have also evaluated the peptide-membrane interaction in biological settings. All peptides showed a decrease in R for erythrocytes and PBMCs, as indicated in Figure 3C,D. This decrease as a function of peptide concentration can be fitted by a hyperbolic curve.

Data were analyzed according to a single binding site model [32]. This enabled us to calculate the apparent dissociation constants (K_d_) presented in Table 3. The K_d_ values showed comparable affinities for cholesterol vs. tocopherol-derived peptides, displaying a higher binding for erythrocytes. The bis-tagged peptides display increased affinity, as it can be seen by a lower K_d_ (one order of magnitude), supporting the previous idea that the mode of interaction is exclusive to the lipid portion of the peptide. DMSO, cholesterol and tocopherol were also tested as controls, but no changes in dipole potential were observed (data not shown).

### 3.3. Lipid-Conjugated Peptides Self-Assemble

**Figure 4:**
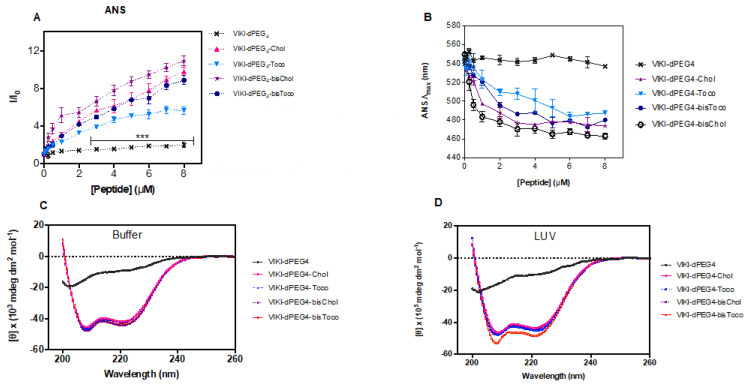
Concentration-dependent aggregation of the VIKI peptides. (**A**) Peptide aggregation in aqueous solution was evaluated by ANS (12.8 μM) fluorescence emission (λ_exc_ = 369 nm; emission spectra integrated from 400 to 600 nm). The values are means ± SEM of at least three experiments. Statistical analysis was performed using a 2-way ANOVA test (*** *p* < 0.001). (**B**) The fluorescence emission maximum wavelength was plotted as a function of peptide concentration. Secondary structure of the VIKI-derived peptides determined by CD: spectra were obtained for VIKI-dPEG_4_ (20 μM), VIKI-dPEG_4_-Chol (20 μM), VIKI-dPEG_4_-Toco (20 μM), VIKI-dPEG_4_-bis-Chol (20 μM), and VIKI-dPEG_4_-bis-Toco (13 μM), in phosphate buffer pH 7.4, at 25 °C, in the presence of (**C**) 2 μM DM detergent, and (**D**) 2 mM POPC LUV. All values are means from at least three independent experiments.

Titration of ANS with VIKI peptides at pH 7.4 (150 mM NaCl) caused an increase in the fluorescence intensity of ANS (Figure 4A) and a significant blue shift of its emission maxima, as shown in Figure 4B, which is indicative of the presence of peptide aggregates in solution. Spectral maxima shift of approximately 80 nm was observed for concentrations above 4 μM. ANS is an environment-sensitive probe. It is essentially non-fluorescent in aqueous media, and becomes fluorescent when bound to hydrophobic sites in aggregates, such as those of proteins and peptides, leading to an increase in the quantum yield [39,40]. In parallel, ANS also undergoes a blue shift and, for this reason, it is commonly used to evaluate the presence of hydrophobic pockets. Since four of the peptides under study have been conjugated with different lipid moieties, they may be prone to aggregation in aqueous solution. When comparing the different lipid conjugations, all peptides induced aggregation. Nevertheless, tocopherol induced the least aggregation when compared with cholesterol. Consequently, the lipid moieties seem to induce peptide aggregation, and cholesterol produces more aggregation than tocopherol, which is even more pronounced when combined with the bis-tagging strategy.

We further assessed whether chemical conjugation with highly hydrophobic molecules such as cholesterol and tocopherol would lead to peptides’ self-assembling in the solution. Having this in mind, we analyzed peptides’ dimensions using dynamic light scattering (DLS). This technique is used for size measurements of nanoscale particles in solution or suspension, and is suitable for peptide/protein assays. The polydispersity index for the lipid-conjugated peptides was below 0.7. Nevertheless, there was an indication that samples had a broad size distribution and do not have a tendency to further aggregate over time. The number-averaged size distribution profiles of each peptide were used to recover the hydrodynamic diameter (D_H_). The lipid-conjugated peptides exhibited a reproducible number-averaged particle D_H_ between 15 and 22 nm, in contrast to VIKI-dPEG_4_, which displayed higher size irrespective of the peptide concentration used (Table 4). The results suggest that the lipid-conjugated peptides self-assemble into stable structures in solution. It is known that self-assembling properties could have implications for the peptides’ antiviral mode of action. The VIKI bis-tagged peptides displayed a number-averaged particle D_H_ between 17 to 22 nm. Surprisingly, the untagged peptides demonstrates higher sizes that increased if the sample was sonicated before DLS measurement, which may be indicative of loose aggregates with an unspecific structure. These observations strongly suggest that the lipid-tagged VIKI peptides tend to self-assemble into stable oligomers, which form confined hydrophobic sites for ANS incorporation.

### 3.4. Lipid-Conjugation Facilitates Folding and Stabilizes Peptide Structure

Several systems have been developed to satisfy the hydrophobic nature of transmembrane segments in proteins, while bringing loop regions into contact with an aqueous phase [41], allowing the use of several biophysical techniques. As membrane proteins, lipid-conjugated peptides usually are not soluble in an aqueous solution. The need for these peptides to reside in microenvironments that satisfy their high hydrophobicity requires special synthetic systems for in vitro work. Consequently, micelles of DM detergent (non-ionic surface) were used to stabilize the peptides in aqueous solution and enable the use of CD spectroscopy to assess the peptides’ secondary structure. The conformation of the VIKI-derived peptides in DM, in the presence or absence of 2 mM POPC vesicles, was analyzed by CD spectroscopy. Two sets of experiments were carried out. In the first set, we recorded the CD spectra of 20 μM peptide solutions in phosphate buffer pH 7.4, in the presence of 2 μM DM (below the CMC [41]), as depicted in Figure 4C. We then added 2 mM POPC LUVs (Figure 4D). Changes in the peptides’ secondary structure were monitored, recording spectra immediately after and after 1 h incubation of the peptides with LUVs. No changes in CD spectra were detected after 1 h incubation. CD signatures of all lipid-conjugated peptides had a pronounced minimum at 200 nm and a shoulder at 220 nm (Figure 4C,D). These CD spectra are indicative of α-helical conformation, except the VIKI-dPEG_4_ that remained largely as a random coil in all conditions. These data suggest that peptides do not change secondary structure after interaction with vesicles. The size of LUV (as well as other relevant parameters) remained invariant after the addition of DM, as assessed by DLS.

### 3.5. MD Simulations Provide Molecular Insights into the Role of Oligomerization and Peptide-Membrane Interaction

According to CD results, lipid-tagged peptides have very stable α-helical conformations, both in solution and in the presence of POPC LUVs, which contrasts with the untagged peptide, which is mainly unstructured in both conditions. ANS and DLS results also indicate that the lipid moieties, and, in particular, cholesterol, induce the formation of stable oligomers. This led us to hypothesize that the formation of oligomers promoted by the lipid tags helps to stabilize the peptides’ secondary structure. To test this hypothesis, we performed MD simulations of the dimeric and monomeric forms of the VIKI-dPEG_4_-Chol peptide in water and in the presence of a POPC membrane. Five independent replicates were simulated in each condition, to obtain statistically meaningful results. All simulations started from an α-helical conformation. Our aim was to compare the stability of this structure in the simulations performed with the dimer with those performed with the monomer. Although the experimental results indicate that the peptide forms higher order oligomers, the use of dimers was chosen for the sake of simplicity and because these simple additions allow the provision of hints into the effect of oligomerization, which can be extrapolated to more complex assemblies.

**Figure 5:**
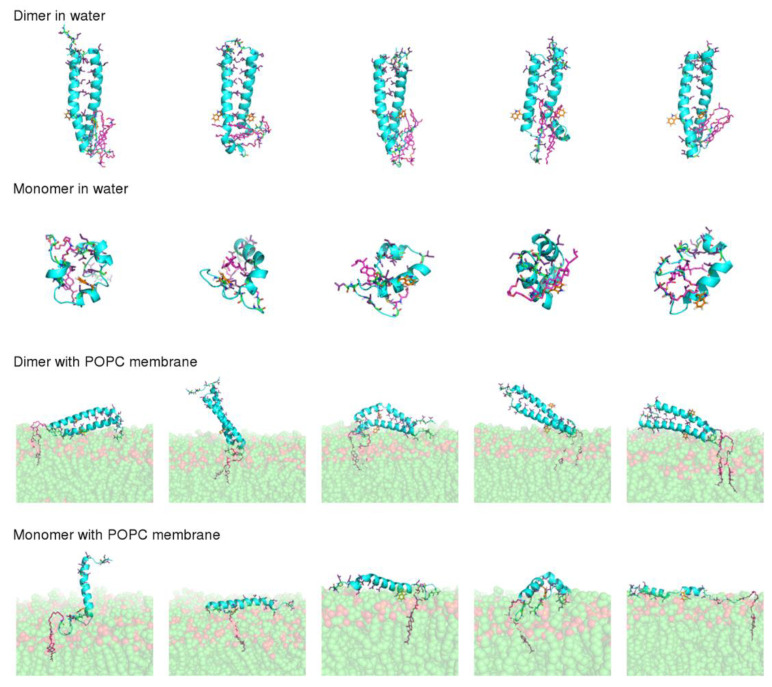
Final conformations of the VIKI-dPEG_4_-Chol peptide after 100 ns of MD simulation. Simulations of the dimeric and monomeric forms of the peptide in water and in the presence of a POPC membrane were performed and each row corresponds to a different simulation set, as indicated in the label. The five structures displayed for each simulation set correspond to independent replicates, with replicates 1 to 5 ordered from left to right. The secondary structure of the VIKI peptide is represented by a cyan cartoon model, the PEG-Chol tail is shown using magenta sticks and the Trp residue is displayed using orange sticks. The hydrophobic residues of the peptide are highlighted using purple sticks and the atoms of the POPC lipids are represented by spheres with the carbon atoms colored in green.

The final conformations obtained after 100 ns of simulation (Figure 5) show that dimerization has a strong effect on peptide stability. In the simulations performed with the dimer, the peptide maintained a straight α-helical conformation, both in water and in the membrane. On the other hand, the monomer was considerably more unstable, particularly in water, where all the replicates adopted more compact structures, with a reduced α-helical content. This result is not surprising given that, in the dimer, the monomers interact through their hydrophobic residues, which become shielded from water, whereas in the monomeric form these residues are exposed when the peptide adopts a straight α-helix conformation, making the structure unstable.

**Figure 6:**
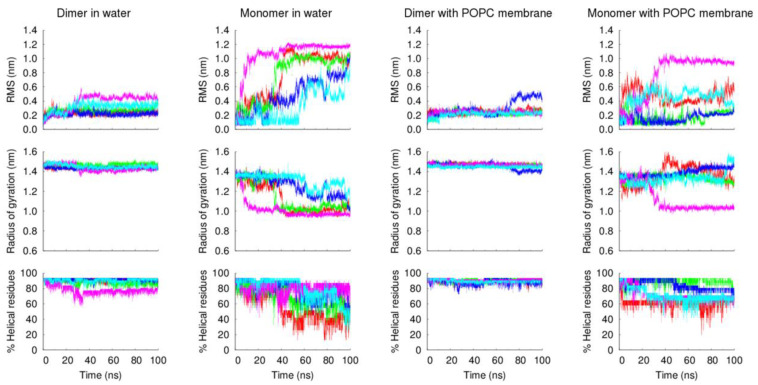
Structural properties of the VIKI-dPEG4-Chol peptide in MD simulations. The temporal evolution of the Cα root mean square deviation (RMSD) from the starting structure, radius of gyration and percentage of α-helical residues (measured by DSSP) is shown for each simulation set. The different lines in each plot correspond to independent replicates: replicates 1 to 5 are colored red, green, blue, magenta and cyan, respectively.

The analysis of the peptides’ structural properties (RMS deviation from the starting structure, radius of gyration and α-helical content), along with the simulation, further confirms the higher stability of the dimeric form of the VIKI-dPEG_4_-Chol peptide, relative to the monomeric form (Figure 6). These and the previous experimental results strongly indicate that the lipid tags induce the formation of oligomers that stabilize the peptide structure.

The MD simulations also explain why the lipid-tagged peptides’ fluorescence emission spectra presented a shift to the blue, relative to the untagged peptide in solution, indicating that the Trp residue of the lipid-tagged peptides is in a more hydrophobic environment. According to the simulations of the dimer in water, the cholesterol tag frequently interacts with the Trp residue, which explains this shift.

Another question that we sought to analyze with these simulations was the molecular details of the VIKI-Chol peptide–membrane interaction. For this analysis, we focused on the simulations performed with the dimer in the presence of a POPC membrane, since the results described above indicate that the peptide most likely forms oligomers. In agreement with the spectroscopic data previously discussed, in our simulations, the interaction of the peptide with the membrane occurs mainly through the cholesterol moiety, which inserts deeply into the membrane (Figure 5). The peptide itself is not inserted into the membrane, laying on the membrane surface in most replicates. The Trp residue remains mainly exposed to the solvent and, in some cases, it interacts with the lipid head groups. The percentage of time that the Trp residue is in contact with each moiety of the POPC molecule in our simulations is 15.92% and 0.04%, for the phosphate and lipid tail carbons, respectively, showing that this residue does not insert deeply into the membrane, as suggested by the experimental results.

## 4. Discussion

Membrane anchoring strategies, such as peptide lipid conjugation, seem to improve the antiviral efficacy of fusion inhibitor peptides, as supported by previous studies [22,42]. Porotto et al. used this methodology to inhibit cell–cell fusion in paramyxoviruses using cholesterol-tagged peptides [19]. The peptides derived from HRC region of the HPIV3 viral fusion protein are effective inhibitors of HPIV, HeV and NiV fusion and entry into target cells [22,43]. The combination of cholesterol tagging, dimerization and inclusion of PEG spacers resulted in improved antiviral potency and extension of in vivo half-life [22,44]. The results demonstrated the potential for exploiting membrane-anchored inhibitors to target membrane-associated proteins and their in vivo efficacy as fusion inhibitors, in some cases with broad-spectrum antiviral activity. However, these studies have not focused on the molecular determinants responsible for this improved efficacy. Lipid conjugation is also known to modify biophysical properties, including water solubility, self-aggregation propensity and thermal stability [23,24]. Knowing this background, we used biophysical approaches to evaluate four different C-terminal lipid-tagged peptides (with cholesterol, bis-cholesterol, tocopherol and bis-tocopherol) and compared them with the unconjugated analogue, in order to correlate their properties with the peptides’ antiviral efficacy.

ANS fluorescence data presented in Figure 4A demonstrate that all the concentration range assessed in these experiments are above the CMC or critical aggregation concentration (CAC) for the four lipid-tagged peptides. ANS has a very low quantum yield in aqueous solution, making it virtually non-fluorescent. Only upon insertion into a hydrophobic environment will it have a marked increase in its quantum yield. Therefore, the lipid-tagged peptides concentration-dependent increase in ANS fluorescence intensity depicted in Figure 4A for all the concentration range clearly indicates that the peptide micelles/aggregates where ANS molecules insert into are already present even at the lower concentration range.

Regarding the location of VIKI lipid-conjugated peptides at the membrane level, our data are highly consistent in showing that the Trp residue of the peptide does not insert in the bilayer. This was further perceived by the fluorescence spectroscopy analysis. Still, our first working hypothesis was to follow the membrane insertion kinetics and evaluate the best lipid conjugation strategy. We were unable to calculate partition coefficients, which is an indication that the Trp residue is not involved in the peptide–membrane interaction. Nevertheless, by the intrinsic fluorescence properties of the VIKI series in water, detergent monomers and LUV, we were able to detect changes in the spectral center of mass, which indicate a conformational change when the peptides are in the presence of the LUV. It is reasonable to predict that the lipid-conjugated peptides are interacting with the LUV, which is corroborated by the fluorescence red shift when POPC LUV are present. This strongly suggests that a structural conformation change must occur, which further contributes to the Trp residue being more exposed to the aqueous environment. In addition, our fluorescence quenching results also show that in the presence of LUV the calculated K_SV_ is still high, an indication of being sufficiently exposed to being quenched by acrylamide. We found that quenching with lipophilic probes was only possible with 16-NS, which, at a first glance, may seem to be a contradiction. However, the 16-NS quencher group has a broader transversal distribution in the membrane than the same moiety in 5-NS, with some of the 16-NS quencher groups transiently locating close to the lipid–water interface, which can explain the quenching results for 16-NS by a well-studied phenomenon called 16-NS snorkeling [26]. The results obtained by MD simulations helped to shed light into these experimental observations. According to the simulations, only the lipid moiety inserts into the membrane bilayer, whereas the rest of the peptide-conjugate remains at the membrane surface, exposing the Trp residue. The fluorescence red shift observed in the presence of POPC LUV can be explained by the fact that the lipid tag can interact with the Trp residue when the peptide is in solution, shielding this residue. On the other hand, in the presence of a membrane, the lipid tag inserts into the bilayer, avoiding the interaction with the Trp residue, which becomes more exposed.

In order to further confirm if a direct interaction between the lipid-conjugated peptides with membranes does occur, we were able to complement our previous approaches by sensing the dipole potential perturbation using the lipophilic probe di-8-ANEPPS and avoiding potential artifacts associated with the peptides’ intrinsic fluorescence. For cell studies, isolated PBMCs and erythrocytes were label with di-8-ANEPPS. Our results suggest a slight preference for cholesterol-enriched membranes. The apparent dissociation constants are similar for cholesterol and tocopherol-tagged peptides. Nevertheless, the K_d_ that recovered from the assays with erythrocytes for the bis-tagged peptides are approximately half of those obtained for VIKI-dPEG_4_-Chol and VIKI-dPEG_4_-Toco, indicating a higher binding of these peptides to the cell membrane.

The lipid conjugation stabilizes the peptide α-helical secondary structure, as the lipid-conjugated peptides exhibited a considerable elevation in their aqueous α-helical content relative to VIKI-dPEG_4_, as measured by CD spectroscopy. MD simulations show that this stabilization is due to the formation of stable oligomers formed by the lipid-tagged peptides. The simulations show that, in the dimeric form, the peptide conjugates are considerably more stable than their monomer counterparts, since the hydrophobic residues can form inter-monomer interactions, stabilizing the α-helical conformation, as commonly observed in α-helical coiled coils [45].

We hypothesize that the VIKI-dPEG_4_ in vitro and in vivo fusion inhibitor potency enhancements are associated with the stable α-helical conformation of the peptides, which, in combination with the lipid conjugation strategy, targets the peptides to the membrane and favors the interaction with the antiviral F protein by optimizing peptide’s structure–function. This interpretation further supports the prior application of lipid conjugation as an adequate strategy for improving the peptides’ pharmacokinetics properties. Future studies should include follow-up work, aiming at a promising candidate for future clinical application as viral entry inhibitors.

## Figures and Tables

**Table 1:** Quenching of VIKI peptides by acrylamide in the absence (buffer) and presence of lipid vesicles. LUV of POPC and POPC:Chol were used at a lipid concentration of 0.5 mM. The Stern–Volmer constants (mM^−1^) were obtained by fitting Equation (3) to the experimental data. Data are presented as mean values ± standard error (SE) from three independent experiments.

			K_sv_ (mM^−1^)		
System	VIKI-dPEG_4_	VIKI-dPEG_4_-Chol	VIKI-dPEG_4_-Toco	VIKI-dPEG_4_-bisChol	VIKI-dPEG_4_-bisToco
Buffer	17.9 ± 0.5	10.0 ± 0.6	9.9 ± 0.6	7.1 ± 0.3	11.4 ± 0.7
POPC	12.7 ± 0.2	8.0 ± 0.3	8.4 ± 0.2	7.1 ± 0.2	6.8 ± 0.1
POPC: Chol (2:1)	12.4 ± 0.5	7.9 ± 0.3	6.1 ± 0.5	7.3 ± 0.4	9.3 ± 0.4

**Table 2:** Stern–Volmer constants (K_SV_) and static constants (V) obtained for the peptides in POPC LUV, quenched by 16-NS, using Equation (4) (Stern–Volmer plots with positive deviation) to fit to the experimental data. Data are presented as the best-fit value from three independent experiments ± SE.

Parameter (mM^−1^)	VIKI-dPEG4	VIKI-dPEG4-Chol	VIKI-dPEG4-Toco	VIKI-dPEG4-bisChol	VIKI-dPEG4-bisToco
K_SV_	~0	1.1 ± 0.3	1.4 ± 0.3	1.3 ± 0.2	0.7 ± 0.2
V	~0	1.4 ± 0.6	1.0 ± 0.5	0.6 ± 0.4	2.7 ± 0.6

**Table 3:** Peptide–cell membrane interaction parameters assessed by di-8-ANEPPS fluorescence. Data are presented as the best-fit value from three independent experiments ± SE.

Peptide	Erythrocytes		PBMCs	
	K_d_ (μM)	R_min_	K_d_ (μM)	R_min_
VIKI-dPEG_4_-Chol	0.13 ± 0.03	−0.33 ± 0.01	0.30 ± 0.12	−0.23 ± 0.02
VIKI-dPEG_4_-Toco	0.14 ± 0.03	−0.30 ± 0.01	0.51 ± 0.12	−0.29 ± 0.02
VIKI-dPEG_4_-bisChol	0.07 ± 0.02	−0.36 ± 0.01	0.22 ± 0.05	−0.24 ± 0.01
VIKI-dPEG_4_-bisToco	0.09 ± 0.03	−0.22 ± 0.01	0.18 ± 0.04	−0.27 ± 0.01
VIKI-dPEG_4_	~0	~0	~0	~0

**Table 4:** DLS number-average hydrodynamic diameter for the peptide aggregates at 20 μM, in PBS buffer, at 25 °C. Data are presented as mean ± SE.

	VIKI-dPEG_4_	VIKI-dPEG_4_-Chol	VIKI-dPEG_4_-Toco	VIKI-dPEG_4_-bisChol	VIKI-dPEG_4_-bisToco
**D_H_ (nm)**	66.3 ± 24.0	14.8 ± 1.0	16.5 ± 1.3	17.0 ± 2.2	22.4 ± 5.6

## Data Availability

Not applicable.
